# Incidence and determinants of perinatal mortality in five urban hospitals in Dar es Salaam, Tanzania: a cohort study with an embedded case–control analysis

**DOI:** 10.1186/s12884-023-06096-1

**Published:** 2024-01-13

**Authors:** Brenda Sequeira Dmello, Thomas Wiswa John, Natasha Housseine, Dan Wolf Meyrowitsch, Jos van Roosmalen, Thomas van den Akker, Monica Lauridsen Kujabi, Charles Festo, Daniel Nkungu, Zainab Muniro, Idrissa Kabanda, Rukia Msumi, Luzango Maembe, Mtingele Sangalala, Ester Hyera, Joyce Lema, Scolastica Bayongo, Johnson Mshiu, Hussein Lesio Kidanto, Nanna Maaløe

**Affiliations:** 1 Comprehensive Community Based Rehabilitation in Tanzania (CCBRT), P. O Box 23310, Dar Es Salaam, Tanzania; 2 Medical College, East Africa, Aga Khan University, Dar es Salaam, Tanzania; 3 Department of Public Health, Global Health Section, University of Copenhagen, Copenhagen, Denmark; 4 Athena Institute, VU University, Amsterdam, the Netherlands; 5 Department of Obstetrics and Gynaecology, Leiden University Medical Center, Leiden, the Netherlands; 6 Ifakara Health Institute, Dar es Salaam, Tanzania; 7 Regional Referral Hospital Dar Es Salaam, Ministry of Health, Dar es Salaam, Tanzania; 8 Presidents Office, Regional and Local Government, Municipal Maternity Hospitals Ubungo and Temeke, Dar es Salaam, Tanzania; 9 Muhimbili Medical Research Center, National Institute for Medical Research, Dar es Salaam, Tanzania; 10 Department of Obstetrics and Gynecology, Copenhagen University Hospital - Herlev Hospital, Herlev, Denmark

**Keywords:** Stillbirths, Neonatal deaths, Perinatal deaths, Dar es Salaam, Tanzania, CCBRT, Urban health, Quality of care, PartoMa, Hypertensive disorders in pregnancy

## Abstract

**Introduction:**

Tanzania has one of the highest burdens of perinatal mortality, with a higher risk among urban versus rural women. To understand the characteristics of perinatal mortality in urban health facilities, study objectives were: I. To assess the incidence of perinatal deaths in public health facilities in Dar es Salaam and classify these into a) pre-facility stillbirths (absence of fetal heart tones on admission to the study health facilities) and b) intra-facility perinatal deaths before discharge; and II. To identify determinants of perinatal deaths by comparing each of the two groups of perinatal deaths with healthy newborns.

**Methods:**

This was a retrospective cohort study among women who gave birth in five urban, public health facilities in Dar es Salaam. I. Incidence of perinatal death in the year 2020 was calculated based on routinely collected health facility records and the Perinatal Problem Identification Database. II. An embedded case–control study was conducted within a sub-population of singletons with birthweight ≥ 2000 g (excluding newborns with congenital malformations); pre-facility stillbirths and intra-facility perinatal deaths were compared with ‘healthy newborns’ (Apgar score ≥ 8 at one and ≥ 9 at five minutes and discharged home alive). Descriptive and logistic regression analyses were performed to explore the determinants of deaths.

**Results:**

A total of 37,787 births were recorded in 2020. The pre-discharge perinatal death rate was 38.3 per 1,000 total births: a stillbirth rate of 27.7 per 1,000 total births and an intra-facility neonatal death rate of 10.9 per 1,000 live births. Pre-facility stillbirths accounted for 88.4% of the stillbirths. The case-control study included 2,224 women (452 pre-facility stillbirths; 287 intra-facility perinatal deaths and 1,485 controls), 99% of whom attended antenatal clinic (75% with more than three visits). Pre-facility stillbirths were associated with low birth weight (cOR 4.40; (95% CI: 3.13-6.18) and with maternal hypertension (cOR 4.72; 95% CI: 3.30-6.76). Intra-facility perinatal deaths were associated with breech presentation (aOR 40.3; 95% CI: 8.75-185.61), complications in the second stage (aOR 20.04; 95% CI: 12.02-33.41), low birth weight (aOR 5.57; 95% CI: 2.62-11.84), cervical dilation crossing the partograph’s action line (aOR 4.16; 95% CI:2.29-7.56), and hypertension during intrapartum care (aOR 2.9; 95% CI 1.03-8.14), among other factors.

**Conclusion:**

The perinatal death rate in the five urban hospitals was linked to gaps in the quality of antenatal and intrapartum care, in the study health facilities and in lower-level referral clinics. Urgent action is required to implement context-specific interventions and conduct implementation research to strengthen the urban referral system across the entire continuum of care from pregnancy onset to postpartum. The role of hypertensive disorders in pregnancy as a crucial determinant of perinatal deaths emphasizes the complexities of maternal-perinatal health within urban settings.

**Supplementary Information:**

The online version contains supplementary material available at 10.1186/s12884-023-06096-1.

## Introduction

Globally, around two million stillbirths and 2.5 million neonatal deaths occurred in 2015, with wide disparities in perinatal mortality between and within low- and middle-income countries (LMICs) and high-income countries (HICs) [1–3]. Stillbirth and neonatal mortality rates in sub-Saharan Africa are more than eight times higher than those in HICs [1–3]. While global perinatal mortality has reduced considerably in recent decades, this reduction still lags markedly behind that of under-five mortality. Stillbirth reduction is particularly slow [1–3]. Furthermore, systematic reviews report an increase in maternal deaths and stillbirths during the COVID-19 pandemic [4]. Importantly, while facility births have increased remarkably, perinatal survival has not followed suit and there is an urgent need for improved quality of maternal and perinatal healthcare [5].

Tanzania has a perinatal mortality of 39.5 per 1,000 total births compared to 34.5 per 1,000 total births in the rest of the Eastern African region [6]. Despite facility-based births in Tanzania's urban areas exceeding 95%, the national stagnation in hospital-based neonatal mortality rates persists alongside a higher incidence of neonatal deaths in urban areas compared to rural areas [7, 8]. Based on Demographic Health Survey data from 2015–2016, an urban perinatal mortality rate was reported of 56.6 per 1,000 total births as compared to 35.9 per 1,000 total births in rural areas and an urban neonatal mortality rate of 39.8/1,000 pregnancies as compared to 21.9/1,000 in rural areas [9, 10]. More specifically, Tanzania’s largest city, Dar es Salaam, comprises a particularly high-burden setting with the magnitude of maternal and perinatal deaths being considerably higher compared to other regions of the country [11–13]. Trends towards similar urban disadvantages are reported from other LMICs [10]. The need is clear: a new understanding of underlying causes and how to improve is warranted to accelerate progress in maternal and perinatal health for people living in urban areas [14].

Pathophysiological causal pathways for antepartum stillbirths in late pregnancy often involve impaired placental function, associated with fetal asphyxia, growth restriction and hypertensive disorders in pregnancy [5, 15]. The most vulnerable period for intrapartum stillbirths and neonatal deaths is the day of birth, with birth asphyxia, prematurity and perinatal sepsis being the leading causes [16, 17]. Poor diagnostics and record keeping, however, often challenge cause-analyses in LMICs and result in causes being classified as “unknown” [18]. Despite this paucity of data, it is clear that perinatal deaths in LMICs can be prevented with basic maternity care [19, 20].

Against this background, the objectives of the present study were : I. to assess the incidence of perinatal deaths in five overcrowded public maternity units in Dar es Salaam and classify these into a) pre-facility stillbirths (absence of fetal heart tones on admission to the study health facility) and intra-facility perinatal deaths (intrafacility stillbirths and pre-discharge early neonatal deaths, where fetal heart tones were heard on admission); and II. To identify determinants of perinatal deaths by comparing the two groups of perinatal deaths to controls (healthy newborns discharged home alive). The ultimate purpose was to inform and strengthen the ongoing quality improvement initiatives for maternal and perinatal care in which this study is embedded (The PartoMa project and the CCBRT-Dar es Salaam regional maternal and newborn healthcare strengthening program) [17, 18].

## Methods

### Setting

The study was carried out in the five busiest government hospitals in Dar es Salaam, Tanzania. In these five hospitals, 60–70% of all births in the city are estimated to take place, and in each health facility (HF) between 6,000 and 10,000 women give birth per year [21]. These HFs are part of a network of 22 HFs in Dar es Salaam region, which collaborate in a maternal and newborn healthcare strengthening program established in 2010 [22]. This program is implemented through a public–private partnership between the regional health authorities and the non-governmental organization ‘Comprehensive Community Based Rehabilitation in Tanzania’ (CCBRT). Furthermore, the five hospitals are the study sites of the PartoMa project, which seeks to achieve best possible maternal and newborn care by adapting clinical guidelines and skills training to the local context [21, 23].

Three of the study HFs are Regional Referral Hospitals (RRHs): HF1, HF2 and HF3. The other two are upgraded health centers that serve as primary level maternity hospitals: HF4 and HF5. All five facilities provide comprehensive care during childbirth that includes vacuum assisted births, cesarean section (CS) and blood transfusions. While the three RRHs have neonatal high care units, the other two (HF4 and HF5) refer all sick newborns to the nearest RRH. All five hospitals receive referrals from dispensaries and health centers throughout the wider Dar es Salaam region. Due to resource-constraints, blood tests for hematology, liver function and renal function are not routinely performed for laboring women with hypertension. All five hospitals typically serve women of lower socio-economic status [24]. Dispensaries and health centers provide a wide range of preventative and first line emergency care, which includes reproductive healthcare, antenatal care (ANC) and care during birth for low-risk pregnant women. Dispensaries are expected to refer all high-risk pregnancies, women with abnormal labor progress, obstetric complications and intrauterine fetal deaths.

**Fig. 1:**
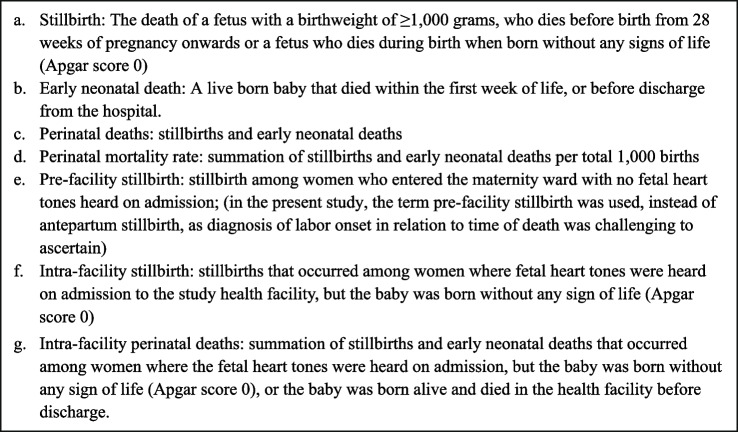
Definition of stillbirths and perinatal deaths in this study, based on the WHO International Classification of Disease and other studies [25, 26, 29]

Figure 1 presents the definitions of stillbirths and early neonatal deaths as defined by the World Health Organization (WHO) [25]. Additional terms used in this study, including pre-facility and intra-facility stillbirth are inspired by previous studies [5, 26]. Definitions e–g were applied instead of antepartum stillbirth, fresh stillbirth and macerated stillbirths due to the low reliability of the stillborn baby’s appearance to the time of fetal death [27, 28].

### Study population

For our cohort study, in order to assess incidence of perinatal deaths, the study population consisted of all births in the five hospitals from 1^st^ January 2020 to 31^st^ December 2020, including all perinatal deaths diagnosed before discharge. For the embedded case-control study, we included a subset of singleton births with birthweight ≥ 2,000 g who had their medical case records available for analysis. Newborns with major congenital malformations were excluded. Within this category, cases of perinatal deaths were sub-categorized by presence or absence of the fetal heart tones on admission to the study HF, as follows:

1. Pre-facility stillbirth, where no fetal heart tones were heard on admission to the maternity ward.
2. Intra-facility perinatal deaths refer to the sum of intra-facility stillbirths and early neonatal deaths where fetal heart tones were heard on admission. For intra-facility stillbirths, the fetus died before birth, and for early neonatal death, the newborn died before discharge**.**

Controls were defined as singleton, live newborns with a birthweight ≥ 2,000 g, Apgar score of ≥ 8 at one minute and ≥ 9 at five minutes, who did not require bag and mask resuscitation and who were discharged home alive [30]. Controls were selected to match the included intra-facility perinatal deaths by month and facility of birth.

### Data collection

For calculation of perinatal mortality rates, we collected data on total births, livebirths, stillbirths, early neonatal deaths, CS, vacuum-assisted births and maternal deaths, which are available in the five hospitals’ routine National Health Information System (MTUHA). Additional information pertaining to perinatal deaths was extracted from the Perinatal Problem Identification (PPIP) database, which contains additional information on actual birthweight, multiple pregnancy and presence of congenital malformations [21].

For the case–control study, all perinatal deaths that met the inclusion criteria were extracted from the PPIP database and their paper-based medical records were intensively searched for. If medical records were retrieved, women were included, and data extracted from their records. Controls were retrieved from the piles of medical records (mainly partographs) of all women with livebirths, which were separated by month of birth and divided into vaginal and cesarean births. The average CS rate in the study sites (2019 data) varied from 18 to 25% [21]. The ratio of vaginal to cesarean births was approximately 4:1. In accordance with the sample size calculation presented below, and by use of a random number generator, controls were then systematically sampled by inclusion of every 10^th^ file, with each fifth of the included files being selected from the CS pile. When the selected controls did not meet the inclusion criteria, the next 10^th^ file was selected.

A data collection tool was developed on Open Data Kit (ODK) XForm using a data dictionary and deployed to an ODK app on smart devices (www.betterevaluation.org). Validation codes were incorporated in the tool to limit data errors and the tool was pilot tested in each study facility by BSD. Data extraction from files was performed by midwives and medical doctors working in the study sites who had all attended a three-day data entry training, conducted by BSD. The first 50 files were double entered by TWJ to check accuracy of data entry. Any discrepancy in double entry was re-checked. BSD reviewed data entry progress and related quality every week throughout the data collection period. All files were assigned a unique identification number and personal identifiers removed. The study database was stored in Ifakara Health Institute Data center, Tanzania.

### Sample size calculation for case-control study

The sample size was estimated using an alpha of 0.05, power of 0.8, ratio of cases to control of 1:5 and assuming an odds of intra-facility perinatal deaths of 1.45. The estimated sample was 395 cases for intra-facility deaths. However, in this study we obtained only 287 cases for intra-facility deaths.

### Variables of interest for the case control study

**Fig. 2:**
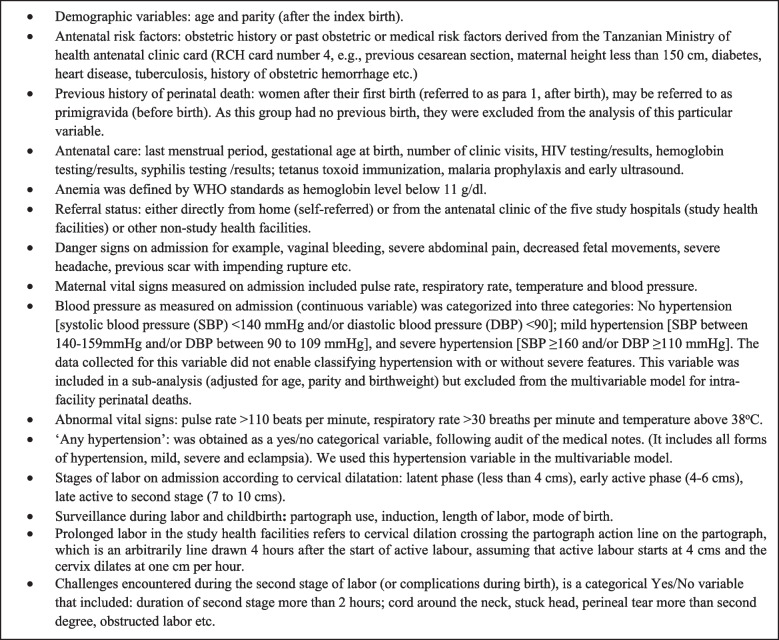
Variables of interest included in the embedded case-control study

For the case-control study, the variables of interest are presented in Fig. 2 and Table 3. These variables were selected from the literature to broadly assess antenatal and admission risk factors as well as intrapartum quality of care provision, including surveillance and associated treatment if needed related to maternal vital signs, fetal heart rate and labor progress [31]. Notably, the selection of variables was limited to what could retrospectively be assessed in the case files.

### Data management and analyses

Data cleaning and analysis was performed using Stata version 14.2 (Stata Corp, Texas, USA). Categorical data were summarized using frequencies and percentages while continuous data were summarized using mean and standard deviation. Stillbirth rate and perinatal mortality were obtained using total births from the five study HFs in the year 2020 per 1,000 total births. A Pearson chi-square test was used to determine associations between variables of interest and outcome variables. One-way ANOVA test was used to compare mean maternal age of the three groups in the case control study population (healthy women, pre-facility stillbirths and intra-facility perinatal deaths). Both bivariable and multivariable logistic regressions were performed to identify the presence of a significant association between independent variables and the dependent variable. Variables with a *p*-value of < 0.2 were considered for multivariable analysis. Any variable whose univariable test has a *p*-value < 0.25 along with all variables of known clinical importance should be included into multivariable analysis [32]. Furthermore, variables with a high proportion of missing cases above 10% were omitted from the multivariable analysis. Following fitting of the model, we assessed the importance of each covariate using p-values. Variables that did not contribute at traditional levels of statistical significance (*p* ≥ 0.05) were eliminated. Bivariate analyses were used for pre-facility stillbirths and multivariable analyses were used for analyses of intra-facility perinatal deaths. Crude Odds Ratio (cOR) and adjusted Odds Ratio (aOR) were calculated with 95% confidence interval (CI). Free text descriptions in women’s files on the care given in response to abnormal vital signs were manually extracted from the notes, categorized and presented. Results were presented using tables and figures.

## Results

From 1 January 2020 to 31 December 2020, the five public HFs in Dar es Salaam registered a total of 37,787 births. During this year, the perinatal death rate was 38.3 per 1,000 total births (1447 deaths). The stillbirth rate was 27.7 per 1,000 total births (1,048 stillbirths) of which 926 (88.4%) were pre-facility stillbirths and 122 (11.6%) intra-facility stillbirths. The pre-discharge neonatal death rate was 10.9 per 1,000 live births (399 neonatal deaths) (Table 1).

**Table 1:** Description of total births, perinatal deaths, maternal deaths, cesarean births, and vacuum assisted births in five urban health facilities in Dar es Salaam in 2020^a^

	**Total**	**Regional Referral Hospitals**			**Primary Maternity Hospitals**	
	ALL	HF1	HF2	HF3	HF4	HF5
Total Births (TB)	**37,787**	5,803	8,384	5,826	10,337	7,437
Live Births (LB)	**36,758**	5,617	8,128	5,589	10,127	7,297
Cesarean section births (n)	**10,375**	2,118	2,043	2,406	2,211	1,597
Cesarean section rates (%)	**27.5**	36.5	24.4	41.3	21.4	21.5
Vacuum assisted births (n)	**934**	121	355	151	177	130
Vacuum assisted rates (%)	**2.5**	2.1	4.2	2.6	1.7	1.7
Neonatal deaths (1)	**399**	92	179	74	26	28
Stillbirths (2)	**1,048**	186	256	237	210	159
Perinatal deaths (sum of 1 and 2)	**1,447**	278	435	311	236	187
Maternal deaths	**42**	9	7	9	15	2
Neonatal death rate/1000 LB	**10.9**	16.4	22.0	13.2	2.6	3.8
Stillbirth rate/1000 TB	**27.7**	32.1	30.5	40.7	20.3	21.4
Perinatal death rate/1000 TB	**38.3**	47.9	51.9	53.4	22.8	25.1
Maternal Mortality Ratio/100000 LB	**114.3**	160.2	86.1	161.0	148.1	27.4

*HF* Health facility

^a^From Facility birth registers (MTUHA 12)

**Fig. 3:**
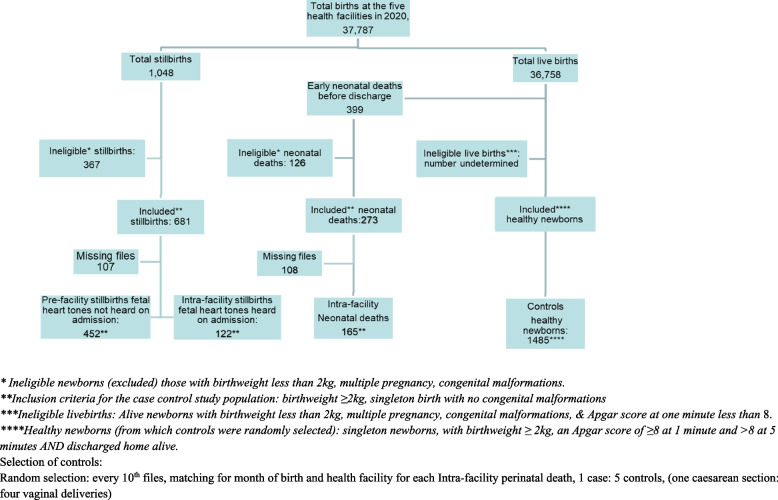
Flow chart for selection of the case-control study population

As a subset of this population, the case–control study included 2,224 women with singleton births and with birthweights equal to or above 2000 g. Of the 1,048 stillbirths, 681 (65.0%) were eligible for inclusion, of which, 574 medical files (84.3%) were retrieved. Among the retrieved files, 452 were pre-facility stillbirths and 122 were intra-facility stillbirths. Of the 399 early neonatal deaths, 273 (68.4%) fulfilled the inclusion criteria, of which we retrieved 165/273 (60.4%) medical files for the in-depth review. As a result, the case control study included 452 pre-facility stillbirths; 287 intra-facility perinatal deaths (122 intra-facility stillbirths plus 165 intra-facility neonatal deaths); and 1,485 controls (a total of 2,224 women) (Fig. 3).

The case-control study’s findings are presented in the sections below. Characteristics of the women in the study population are presented in Table 2. The factors associated with perinatal deaths are described in detail in Table 3. For reference, additional descriptive data are presented in Supplementary Tables 1, 2 and 3.

**Table 2:** Background characteristics in the case–control population, 2,224 singleton pregnancies with birthweight ≥ 2000 g, (excluding congenital malformations) in five urban health facilities in Dar es Salaam

Variables	Total n (%)	Healthy babies n (%)	Pre-facility stillbirths n (%)	Intra-facility perinatal deaths n (%)	*p-*value
**Total**	2,224	1,485 (66.8)	452 (20.3)	287 (12.9)	
**Age groups (years)**					
15–19	211 (9.5)	164 (11.0)	25 (5.5)	22 (7.7)	** < 0.001**
20—35	1,760 (79.1)	1,174 (79.1)	361 (79.7)	225 (78.4)	
36—45	253 (11.4)	147 (9.9)	66 (14.6)	40 (13.9)	
**Mean Age (SD)**	27 (6.2)	26.5 (6.1)	28.2 (6.3)	27.5 (6.3)	** < 0.001**
**Parity after current birth**					
Para 1	893 (40.2)	625 (42.1)	153 (33.8)	115 (40.1)	**0.009**
Para 2—4	1,197 (53.8)	775 (52.2)	262 (57.9)	160 (55.7)	
Para ≥ 5	134 (6.03)	85 (5.7)	37 (8.2)	12 (4.2)	
**Gestation age (weeks)**					
< 37	305 (13.7)	119 (8.0)	143 (31.6)	43 (14.9)	** < 0.001**
37–40	1,022 (45.9)	751 (50.6)	150 (33.2)	121 (42.2)	
> 40	721 (32.4)	514 (34.6)	116 (25.7)	91 (31.7)	
Missing information	176 (7.9)	101 (6.8)	43 (9.5)	32 (11.2)	
**Antenatal clinic attendance**					
Never attended	8 (0.4)	2 (0.1)	3 (0.7)	3 (1.0)	** < 0.001**
1–3 visits	518 (23.3)	317 (21.3)	153 (33.8)	48 (16.7)	
4–6 visits	1,471 (66.1)	1,021 (68.8)	260 (57.5)	190 (66.2)	
> 6 visits	156 (7.0)	115 (7.7)	23 (5.1)	18 (6.2)	
Missing information	71 (3.2)	30 (2.0)	13 (2.9)	28 (9.7)	
**Past history of perinatal death**					
Yes-previous perinatal death	194 (14.6)	135 (15.7)	47 (15.7)	12 (7.0)	**0.001**
No previous perinatal death	1,137 (85.4)	725 (84.3)	252 (84.3)	160 (93.0)	
Para 1 (First pregnancy-excluded)	893 (40.2)	625 (42.1)	153 (33.9)	115 (40.1)	

*• due to rounding to one decimal place, the total percentages may not add up to exactly 100%*

**Table 3:** Determinants of perinatal death in the case-control study’s 2224 singleton pregnancies with birthweight ≥ 2000 grams in five urban health facilities in Dares Salaam (excluding congenital malformations)

	**Total**	**Healthy babies**	**Pre-facility stillbirths**^**a**^	**Intra-facility perinatal deaths**^**b**^	**Bivariate logistic regression (compared to healthy babies)**	**Multivariable logistic regression (compared to healthy babies)**
	*N*=2,224 (%)	*N*=1,485 (%)	*N*=452 (%)	*N*=287 (%)	*Pre-facility stillbirths	**Intra-facility perinatal deaths
**Age (years)**					cOR (95%CI)	aOR (95%CI)
15- 19 years	211 (9.5)	164 (11.0)	25 (5.5)	22 (7.7)	**0.50 (0.32-0.77)**	0.45 (0.18-1.13)
20 - 35 years	1,760 (79.1)	1,174 (79.1)	361 (79.7)	225 (78.4)	1^^^	1^^^
36 - 45 years	253 (11.4)	147 (9.9)	66 (14.6)	40 (13.9)	1.46 (1.07-2.02)	0.89 (0.42-1.89)
Missing information	0	0	0	0		
**Parity**						
Para 1	893 (40.2)	625 (42.1)	153 (33.8)	115 (40.1)	0.72 (0.58-0.91)	1.02 (0.60-1.76)
Para 2-4	1,197 (53.8)	775 (52.2)	262 (57.9)	160 (55.7)	1^^^	1^^^
Para ≥ 5	134 (6.0)	85 (5.7)	37 (8.2)	12 (4.2)	1.29 (0.85-1.94)	0.99 (0.37-2.65)
Missing information	0	0	0	0		
**Referral status**						
Self-referred from home	1,807 (81.3)	1,281 (86.3)	308 (68.1)	218 (75.9)	0.53 (0.35-0.80)	0.86 (0.30-2.48)
Study HFs^c^	134 (6.0)	77 (5.2)	35 (7.7)	22 (7.7)	1^^^	1^^^
Peripheral HFs (lower-level HFs)	283 (12.8)	127 (8.5)	109 (24.1)	47 (16.4)	**1.89 (1.17-3.03)**	1.05 (0.32-3.47)
Missing information	0	0	0	0		
**Antenatal risk factor**^d^						
No	1,930 (86.8)	1,346 (90.7)	354 (78.3)	230 (80.1)	1^^^	1^^^
Yes	294 (13.2)	139 (9.4)	98 (21.7)	57 (19.9)	**2.68 (2.02-3.56)**	3.70 (1.96-6.98)
**Admission danger sign documented**^e^						
None	1,720 (77.3)	1,315 (88.5)	200 (44.2)	205 (71.4)	1^^^	1^^^
Yes-danger sign	504 (22.7)	170 (11.4)	252 (55.7)	82 (28.6)	**9.75 (7.63-12.45)**	1.51 (0.76-2.99)
**Any hypertension (hypertensive disorders described as a complication during labor and birth)**^**f**^						
No hypertension	2,059 (92.6)	1,425 (95.9)	377 (83.4)	257 (89.6)	1^^^	1^^^
Any hypertension	165 (7.4)	60 (4.1)	75 (16.6)	30 (10.5)	**4.72 (3.30-6.76)**	**2.90 (1.03-8.14)**
**Stage of labour on admission (according to cervical dilatation)**						
Latent phase or earlier (0-3 cms)	621 (27.9)	387 (26.1)	138 (30.5)	96 (33.4)	1.77 (1.34-2.34)	1.22 (0.68-2.20)
Early active phase (4-6cms)	748 (33.6)	571 (38.4)	115 (25.4)	62 (21.6)	1^^^	1^^^
Late active phase >6 cms	694 (31.2)	494 (33.3)	138 (30.5)	62 (21.6)	1.39 (1.05-1.83)	1.16 (0.65-2.07)
Missing information	161 (7.2)	33 (2.2)	61 (13.5)	67 (23.3)		
**Status of liquor on admission**						
Intact membranes	1,274 (57.3)	929 (62.5)	224 (49.6)	121 (42.2)	1^^^	1^^^
Clear liquor	486 (21.8)	392 (17.6)	44 (9.7)	50 (17.4)	**0.47 (0.33-0.66)**	1.12 (0.65-1.91)
Meconium liquor	125 (5.6)	39 (2.6)	60 (13.3)	26 (9.1)	**6.38 (4.16-9.80)**	**4.44 (1.86-10.57)**
Blood-stained liquor	8 (0.4)	0	8 (1.7)	0		
Missing information	331 (14.9)	125 (8.4)	116 (25.6)	90 (31.3)		
**Prolonged labour, the partograph’s action line crossed**^**g**^						
No	1,738 (78.1)	1,282 (86.3)	314 (69.5)	142 (49.5)	1^^^	1^^^
Yes	168 (7.6)	89 (5.9)	30 (6.6)	49 (17.1)	1.38 (0.89-2.12)	**4.16 (2.29-7.56)**
Missing information	318 (14.3)	114 (7.7)	108 (23.9)	96 (33.4)		
**Induction of labour**						
No induction	2,095 (94.2)	1,443 (97.2)	391 (86.5)	261 (90.9)	1^^^	1^^^
Induction of labour	129 (5.8)	42 (2.8)	61 (13.5)	26 (9.1)	**5.36 (3.56-8.07)**	**2.74 (0.97-7.72)**
**Mode of birth**						
Spontaneous vaginal birth	1,620 (72.8)	1,120 (75.4)	357 (78.9)	143 (49.8)	1^^^	1^^^
Vacuum extraction	28 (1.3)	12 (0.8)	4 (0.9)	12 (4.2)	1.05 (0.34-3.26)	**6.23 (1.65-23.55)**
Caesarean section	533 (24.0)	345 (23.2)	74 (16.4)	114 (39.7)	**0.67 (0.51-0.89)**	0.93 (0.53-1.63)
Breech	33 (1.5)	4 (0.3)	12 (2.7)	17 (5.9)	**9.41 (3.02-29.36)**	**40.3 (8.75-185.61)**
Missing information	10 (0.4)	4 (0.3)	5(1.1)	1 (0.3)		
**Challenges in the second stage of labour**^**h**^						
No challenges documented	1,780 (80.0)	1,400 (94.3)	266 (58.9)	114(39.7)	1^^^	1^^^
Yes	444 (19.7)	85 (5.7)	186 (41.2)	173 (60.3)	**11.52 (8.63-15.36)**	**20.04 (12.02-33.41)**
**Birthweight**						
2000-2499	251 (11.3)	73 (4.9)	122 (27.0)	56 (19.5)	**4.40 (3.13-6.18)**	**5.57 (2.62-11.84)**
2500-3000	683 (30.7)	434 (29.2)	165 (36.5)	84 (29.3)	1^^^	1^^^
3000-3499	829 (37.3)	634 (42.7)	96 (21.2)	99 (34.5)	0.41 (0.30-0.53)	0.76 (0.43-1.35)
3500-3999	355 (16.0)	281 (18.9)	41 (9.1)	33 (11.5)	0.38 (0.26-0.56)	0.44 (0.2-0.94)
Greater than 4000	106 (4.7)	63 (4.2)	28 (6.2)	15 (5.2)	1.17 (0.72-1.89)	1.36 (0.49-3.82)
Missing information	0	0	0	0	-	-
**Description and sub-analysis of Blood pressure measurement on admission** (variable excluded from the main multivariable analysis)^i^						
No hypertension	1,740 (78.2)	1,235 (83.2)	316 (69.9)	189 (65.9)	1^^^	1^^^
Mild hypertension	249 (11.2)	146 (9.8)	72 (16.0)	31 (10.8)	**1.93 (1.42-2.62)**	0.8 (0.36-1.78)
Severe hypertension	71 (3.2)	36 (2.4)	28 (6.2)	7 (2.4)	**3.04 (1.83-5.06)**	1.37 (0.32-5.77)
Missing	164 (7.4)	68 (4.6)	36 (7.9)	60 (21.0)		

Notes: Statistically significant Odds Ratio and 95% confidence intervals have been bolded. Some of the percentages may be over 100% due to rounding up to one decimal place

1^^^refers to the reference sub-group

^*^For the pre-facility stillbirths’ bivariate logistic regression is applied (some variables concern management after death, for instance, prolonged labour, challenges in second stage and mode of birth were intra-facility practices, among women who had no fetal heart tones on admission to the study HF (pre-facility stillbirths). This illustrates that women suffering from intrauterine fetal death prior to admission simultaneously had increased odds for suffering from prolonged labour and challenges in the second stage

^**^For the Intra-facility perinatal deaths, a multivariable logistic regression is presented. Adjusted Odds Ratio (aOR) has been adjusted for age, parity, referral status, antenatal risk factors, admission danger signs, stage of labor on admission, status of liquor on admission, partograph use with labour crossing the action line, induction of labor, mode of birth, çhallenges second stage of labor, baby birthweight, any hypertension during labour or birth

^a^Pre-facility stillbirths- arriving at the health facility (HF), without a fetal heart tone detected

^b^Intra-facility perinatal death includes intra-facility stillbirths and early neonatal deaths before discharge

^c^Admission from the antenatal ward of one of the five study health facilities

^d^Antenatal risk factors obstetric history or past obstetric or medical risk factors derived from the Tanzanian Ministry of health antenatal clinic card (RCH card number 4, e.g., previous caesarean section, previous perinatal death, less than 20 years of age, long birth interval (more than 10 years), Rhesus negative blood group, pelvic deformity, maternal height less than 150 cm, diabetes, heart disease, tuberculosis, history of obstetric hemorrhage etc.); The 294 women, who answered yes to this question, reported more than one risk factor

^e^Danger signs on admission: These include signs of maternal fetal complications (reduced fetal movements, vaginal bleeding, severe headache, blurred vision, severe abdominal pain, fits, or severe body weakness/fainting or fits

^f^Any hypertension was obtained as a yes/no categorical variable, following audit of the medical notes. (It includes all forms of hypertension, mild, severe and nine women with eclampsia). The proportion appears lower than the variable "measurement of hypertension on admission", perhaps due to poor documentation, milder forms of hypertension maybe under recorded in the case-notes as explained in the limitation section)

^g^The action line on the partograph is an arbitrarily line drawn 4 hours after the start of active labour, assuming that active labour starts at 4 cms and the cervix dilates at one cm per hour. In study HFs, cervical dilatation crossing the action line is considered prolonged labor

^h^Challenges during the second stage included duration of second stage more than 2 h; cord around the neck, stuck head, shoulder dystocia, fetal distress, hemorrhage, rupture of uterus, perineal tear more than second degree, obstructed labor

^i^Blood pressure on admission was the actual blood pressure on admission (continuous numeric) that was then categorized into three categories: no hypertension, mild hypertension and severe hypertension. The data collected for this variable did not enable classifying hypertension with or without severe features. A sub-analysis was performed with this variable (adjusted for age, parity and birthweight). This variable was excluded from the main multivariable model for intra-facility perinatal deaths

### Characteristics of the women in the case–control study population

Mean age of the women was 27.0 (SD ± 6.2). There was a small difference in mean age of women between the groups with healthy newborns and with pre-facility and intra-facility perinatal deaths, but no difference between pre-facility and intra-facility perinatal deaths groups. The history of a previous perinatal death among the 194/1,331 (14.6%) multiparous women was similar between controls (135/860; 15.7%) and pre-facility stillbirths (47/299; 15.7%). However, among multiparous women with a past history of previous perinatal death, a lower proportion of intra-facility perinatal deaths was observed (12/172; 7.0%; aOR 0.32; 95% CI: 0.13–0.81) (Supplementary Table 4).

### Description of antenatal care (ANC)

All women in the case–control study, except eight, attended ANC with 1,627/2,153 (75.5%) attending four or more visits (Table 2). More than 90% of the women received routine testing for Human Immunodeficiency Virus (HIV) and syphilis, tetanus immunization and presumptive treatment for malaria. Of all women, 402 (18.1%) had an ultrasound examination, but only 21/402 (5.2%) before 24 weeks of gestation. Among the 1850 (83.1%) women with available hemoglobin test results, mean hemoglobin (Hb) was 10.9 g/dl ± 1.3 SD and severe anemia (Hb ≤ 8 g/dl) occurred in 45 (2.4%) women**.** There was no difference in mean hemoglobin in the three outcome groups (Supplementary Table 1).

### Referral status and management on admission

Among the case control study population, self-referrals from home occurred in 1,807/2,224 (81.3%) women and 283/2,224 (12.7%) were referred from other non-study HFs (mainly health centers and dispensaries). More specifically, among pre-facility stillbirths, 109 (24.1%) were referred from lower-level HFs, and our data did not indicate if the fetal heart tone was present when the women were admitted in the primary-level HF.

While more than 86% of women had their vital signs measured on admission, very few among those with abnormal vital signs had documentation of any action taken in response. For example, a blood pressure measurement on admission was recorded for 2,060 (92.6%) of women. Among the 71 women with severe hypertension (SBP ≥ 160 and/or DBP ≥ 110 mmHg) measured on admission, 16 (22.5%) of their case-notes had specific treatment recorded.

Concerning the stage of labor on admission, 621 (30.1%) were admitted in the latent stage, 748 (36.3%) were admitted in early active labor (cervical dilatation between 4 and 6cms) and 694 (33.7%) were admitted in late active labor with cervical dilation between 7 to 10 cms. There were no statistically significant differences between the groups.

### Surveillance and management during labor and childbirth

We reviewed 2,085 available partographs. All three sections of the partograph (maternal vital signs, labor progress and fetal heart rate) were completely filled in for 874/2,085 (41.9%) women. 

We noted that women with fetal death prior to admission (pre-facility stillbirth) had significantly increased odds of experiencing challenges during the second stage of labor ( cOR 11.52; 95%CI: 8.63-15.36), and breech presentation with vaginal birth (cOR 9.41;95% CI: 3.02-29.36) (Table 3).

CS was performed in 533/2,224 (23.9%) women. Out of the total CSs, 469/533 (88%) were performed as emergencies. The commonest indication for CS was previous CS in 136/533 (25.5%) women, followed by obstructed labor in 103/533 (19.3%) and fetal distress in 70/533 (13.1%). The study design ensured that for the control group, the random selection included CSs that approximated the CS rate in the study hospitals, there were no statistical or clinically relevant differences in CS as mode of birth and birth outcomes. Among the 533 women with CS birth, we noted 188 (35.2%) perinatal deaths, distributed as follows: pre-facility stillbirths (74/452; 16.4%), intra-facility stillbirths (54/122; 44.3%) and intra-facility early neonatal deaths (60/165; 36.4%).

### Maternal birth outcomes

Among our study population, 1,862/2,224 (83.7%) women reported no complications during labor and childbirth, while 412 complications were extracted from the remaining 362 women, some of whom reported more than one complication. Hypertension was reported most frequently (165/412, 40.0%) followed by anemia (69/412, 16.7%) and hemorrhage (51/412, 12.3%). Within the study population, there was one maternal death due to hemorrhage from placental abruption in the intra-facility stillbirth group. Uterine rupture occurred in 18 women, including in six among women with pre-facility stillbirth, 11 women with intra-facility perinatal death, and one woman in the control group.

### Factors associated with increased odds ratio for pre-facility stillbirths

Compared to healthy newborns, factors associated with increased odds of pre-facility stillbirths included the following: any hypertension as a complication during intrapartum care (cOR 4.72; 95% CI: 3.30–6.76); low birth weight (newborns within the lowest included birthweight category between 2,000 and 2,500 g), (cOR 4.40; 95% CI: 3.13–6.18); presence of at least one risk factor detected during ANC (cOR 2.68; 95% CI 2.02- 3.56) and if the pregnant woman was referred from peripheral HFs (cOR 1.88; 95% CI 1.17–3.03) (Table 3).

Furthermore, a sub-analysis of maternal blood pressure measured on admission (adjusted for age of the woman, parity and newborn birthweight), revealed increased odds of pre-facility stillbirths compared to healthy newborns: mild high blood pressure measurement on admission (cOR 1.93; 95% CI: 1.42–2.62); severe high blood pressure measured on admission (cOR 3.04; 95% CI: 1.83–5.06) (Table 3 and Supplementary table 2).

### Factors associated with increased odds ratio for intra‑facility perinatal deaths

Compared to healthy newborns (controls), determinants of intra-facility perinatal death included: breech birth (aOR 40.3; 95% CI: 8.75–185.61); complications in the second stage of labor (aOR 20.04; 95% CI: 12.02–33.41); vacuum-assisted birth (aOR 6.23; 95% CI: 1.6–23.55); low birth weight or late preterm birth with birthweight between 2,000 and 2,500 g (aOR 5.57; 95% CI: 2.62–11.84); cervical dilation that crossed the partograph’s action line (aOR 4.16; 95% CI:2.29–7.56); meconium liquor on admission (aOR 4.44; 95% CI:1.86–10.57); at least one risk factor detected during ANC (aOR 3.7; 95% CI 1.96–6.98) and maternal hypertension during intrapartum care (aOR 2.9; 95% CI 1.03–8.14) (Table 3).

## Discussion

In these five overcrowded maternity hospitals in the rapidly urbanizing city of Dar es Salaam, we found a high overall facility-based perinatal mortality rate of 38.3 per 1,000 births (stillbirth rate 27.7 per 1,000 total births, pre-discharge neonatal death rate 10.9 per 1,000 live births). Of all stillbirths, 88.4% were pre-facility, and this is in an urban context of almost 100% antenatal clinic attendance and above 95% facility birth rates [33].

Among women in the embedded case–control study, the determinants that raised the odds for both pre-facility stillbirths and intra-facility perinatal deaths included: low birth weight and maternal hypertension. Among women with intra-facility perinatal deaths, breech presentation, complications in the second stage of labor, prolonged labor (cervical dilatation crossing the action line on the partograph), presence of any antenatal risk factor (particularly relevant for multiparous women) and meconium-stained liquor were additional significant determinants for perinatal death. These findings are comparable to reports from other low-resource settings [34, 35].

The higher than expected proportion of pre-facility stillbirths stands in contrast to regional estimates, based on which we expected that only half would fall into this category [36]. This may be partially explained by the local referral criterion, requiring pregnant women with intrauterine fetal death to be referred to a secondary level hospital. In the embedded case-control sub-study, however, 68.1% of women with pre-facility stillbirths came directly from home. Our data do not allow conclusions on whether fetal death occurred at home, before labor, at the referring dispensary, during transit to the study HF or while waiting to be assessed and admitted in the study HFs.

While the pre-versus intra-facility proportions differ, the stillbirth rate is similar to that reported in Northern Tanzania [37, 38], but lower compared to facility-based stillbirth rates in referral hospitals in Tanzania: Kilimanjaro Christian Medical Centre (38/1,000 births) and Zanzibar (37.5/1,000 births) [39, 40]. It is however higher compared to rates reported from hospitals in Asia, 16 per 1,000 births in India and 17.6 per 1,000 births in Nepal [41, 42].

The neonatal death rate reported in this study is comparable to the 10.4 per 1,000 births reported previously for 35 zonal, regional and district hospitals in Tanzania [12]. Our findings support emerging evidence for high risk of neonatal and perinatal mortality in hospitals in the densely populated urban areas in Dar es Salaam, which may be similar to other urban centers in East Africa [9, 10, 12].

The high pre-facility stillbirth rate serves as a stark reminder of the imperative to enhance antenatal care to encompass vigilant monitoring and swift response to maternal and fetal complications, especially during the third trimester of pregnancy. This is not only crucial to the study hospitals but also in all referring healthcare facilities [43]. Women with high-risk pregnancies, including those with hypertension require timely diagnosis, more frequent visits, accurate gestational age dating, monitoring of fetal growth and wellbeing, and planned birth often by 39 weeks gestation or earlier when required [44–46]. Notably, we found an excessive number of women with unknown gestational age either due to unknown last menstrual period or missing data, and very few women (5.2%) had access to an early ultrasound. This presents a challenge in planning the time of birth for women with high risk pregnancies or with complications such as hypertension [47]. Also, lack of clarity in the guidelines regarding when to induce labor may have contributed to the high burden of pre-facility stillbirth related to hypertensive disorders [39, 45, 48–53].

Furthermore, we found a strong association between neonates with birthweights between 2,000 to 2,500 g and risks of perinatal death, confirming findings from other studies that low birth weight and prematurity are independent risk factors for poor perinatal outcome [54]. We also recognize that due to the unreliability of gestational age, we could not differentiate co-existent intrauterine fetal growth restriction [55, 56].

 Importantly, pregnant women need quality care throughout the continuum of pregnancy and birth. Increasing the number of visits in the third trimester without strategic investments to address structural gaps in resources and healthcare provider’s skills will add to the workload and is unlikely to reduce the burden of preventable stillbirths and the socioeconomic benefits from lowered mortality [57].

Our study sheds light on the operations of high-volume maternity units in an urban setting where a wide spectrum of both low and high-risk pregnancies are managed, including potentially life-threatening obstetric complications such as hypertension and uterine rupture [34, 58]. This scenario suggests a high-intensity work environment where our study noted that 88% of all CSs were performed as urgent procedures. Within these specific five hospitals, there are notable constraints due to the presence of only one operating theater, high staff turnover, and limited available workforce, which are considerably impeding effective monitoring of women during labor and birth [39]. This situation vividly illustrates the interconnected complex interplay between obstetric complications, infrastructural limitations, insufficient skills, delayed response, adverse perinatal outcomes and an urban healthcare system functioning under immense pressure. Notwithstanding the critical shortage of staff, the findings from our study strongly underscore the pressing necessity to enhance the skills of healthcare providers in managing the second stage of labor, particularly in the case of breech presentation.

Finally, it must be noted that this study was conducted in 2020, where the onset of the COVID-19 pandemic intensified stress on existing fragile low-resource urban health systems [59]. This may have worsened maternity care provision compared to previous years. The COVID-stressor, however, may also be seen as yet another of the many stressors on the urban healthcare system, such as massive urbanization, climate change and political changes. They each, and in combination, expose weaknesses in the provision of maternal healthcare, as here shown, and they require a call for inter-sectoral collaboration to deliver action to ensure safe care for women and children.

### Strengths and limitations

This study includes extensive data on facility births from multiple busy public maternity hospitals that receive women with a mix of low- and high-risk pregnancies, predominantly of lower-socio economic status, and referred from the entire Dar es Salaam region, thereby providing an in-depth understanding of the urban disadvantage experienced by women during pregnancy and childbirth in Dar es Salaam. There are, however, limitations to this study.

This was a retrospective study using data from routine health information systems. Previous studies report that hospital registers may have a high sensitivity and specificity for reporting perinatal outcomes [60, 61]. Furthermore, the Dar es Salaam quality improvement initiative has invested in strengthening routine data at the study HFs since 2010 [21]. Yet, we recognize that the quality of data may not be optimal. We acknowledge that facility-based pre-discharge neonatal mortality, although high, may still be an underestimate, as we could not include neonates who may have died after discharge or referral. We recognize that stillbirths and early neonatal deaths are prone to misclassification, particularly in understaffed facilities where fetal motion, heart rate or respiration in a liveborn infant may not have been observed [3, 26].

Even though we included all eligible cases, the 287 intra-facility perinatal deaths fell short of the 395 required in the initial power calculation. (A proportion of these could not be included due to missing case files). Furthermore, concerning hypertensive disorders, our data and practices in the study HFs did not allow for clinical classification of hypertension with or without severe features. Also, mild cases of hypertension may have been under-reported due to poor documentation. Consequently, the more severe types of hypertensive disorders may more likely have been captured, resulting in selection bias. Lack of storage capacity, poor protection of case notes (particularly during the COVID-19 pandemic), deficient documentation and missing files contributed to shortfall in intra-facility perinatal deaths and may also have resulted in selection bias, which potentially could have resulted in an underestimation of the strength of the associations between the determinants and perinatal deaths. The targeted care for teenagers, grand multiparous women of advanced maternal age and women with previous adverse obstetric outcomes is part of the decade long quality improvement initiative at the five study HFs and may explain the unexpected lower odds for intra-facility deaths in these high-risk groups [22].

We used the term pre-facility stillbirth, where the point of reference was presence or absence of fetal heart sounds on admission [31]. The term pre-facility stillbirth is not entirely synonymous to ‘antepartum stillbirths’ (pre- labor) or ‘macerated stillbirths’, which is based on fetal appearance [5]. Gold et al. consider fetal appearance an unreliable surrogate marker for determining the time of fetal death [28]. These differences in terminology may make comparison of our findings with other studies challenging. Our categorization was the most feasible in the setting and provides a reliable distinction between stillbirths that occurred before or after admission to HFs during labor and birth, but requires accurate fetal heart rate documentation on admission [60].

## Conclusion and recommendations

This study unfolds a high burden of preventable perinatal deaths among urban women in Tanzania with potentially viable newborns in a setting of high antenatal care attendance and high institutional birth rates. The determinants of perinatal death were linked to substandard quality of antenatal and intrapartum care. Context-specific interventions to strengthen the skills and resources for health providers to manage high-risk pregnancies and the second stage of labor are required to address the tremendous burden of perinatal loss in urban maternal health. Further prospective studies that include the wider referral system are recommended to understand and address the complexity of urban perinatal deaths. Each perinatal death is a preventable tragedy requiring urgent mitigation through global, national, regional and city-based prioritization, which has the potential of major socioeconomic return on investment.

### Supplementary Information

**Additional file 1:** **Supplementary Table 1.** Routine antenatal clinic investigations and prophylaxis among 2224 women with singleton pregnancies ≥ 2000gms included in the case-control study population. ** Supplementary Table 2.** Logistic regression for Pre-facility Stillbirths against live healthy newborns. **Supplementary Table 3.** Logistic regression for Intra-facility perinatal deaths (intrafacility stillbirths and early pre discharge neonatal deaths) compared to live healthy newborns.

** Additional file 2.** Perinatal case control study data collection tool.

## Data Availability

The data generated from this study are available in this published article (and the supplementary tables). Additional information is available on request from the corresponding author.

## Abbreviations

LMIC: Low-and-middle income countries
HF: Health facility
HIC: High-income countries
CCBRT: Comprehensive Community Based Rehabilitation in Tanzania
CEmONC: Comprehensive Emergency Obstetric and Newborn Care
CS: Cesarean Section
MTUHA: Mfuma wa Taarifa za Uendeshaji Huduma za Afya—Swahili word referring to the Tanzania national health information system
PPIP: Perinatal Problem Identification Program
WHO: World Health Organization
