# Supplementary material for: Incidence and determinants of perinatal mortality in five urban hospitals in Dar es Salaam, Tanzania: a cohort study with an embedded case–control analysis
Source: BMC Pregnancy Childbirth. 2024 Jan 13;24:62. doi: 10.1186/s12884-023-06096-1 (PMC10787400; doi:10.1186/s12884-023-06096-1)
Supplement: Supplementary file 1 — Additional file 1: Supplementary Table 1. Routine antenatal clinic investigations and prophylaxis among 2224 women with singleton pregnancies ≥ 2000gms included in the case-control study population. Supplementary Table 2. Logistic regression for Pre-facility Stillbirths against live healthy newborns. Supplementary Table 3. Logistic regression for Intra-facility perinatal deaths (intrafacility stillbirths and early pre discharge neonatal deaths) compared to live healthy newborns. [file 12884_2023_6096_MOESM1_ESM.docx]

| **Supplementary Table 1: Routine antenatal clinic investigations and prophylaxis among 2223 women with singleton pregnancies above 2000gms included in the case-control study population** | | | | | |
| --- | --- | --- | --- | --- | --- |
|  | **Total** | **Healthy babies** | **Pre-facility stillbirths** | **Intra-facility perinatal deaths** | **p-value** |
| **Variables** | N=2,224 (%) | N=1,485 (%) | N=452 (%) | N=287 (%) |  |
| **HIV Status** |  |  |  |  |  |
| Positive | 129(5.9) | 87 (6.0) | 27 (6.2) | 15 (5.3) | p= 0.892 |
| Negative | 2,057(94.1) | 1375 (94.1) | 414 (93.9) | 268 (94.7) |  |
| Missing | 38(1.8) | 23 (1.6) | 11 (2.5) | 4 (1.4) |  |
| **Syphilis Status** |  |  |  |  |  |
| Tested Positive | 4(0.2) | 3 (0.2) | 1 (0.2) | 0 | p=0.000 |
| Tested Negative | 2,018 (90.8) | 1,385 (93.3) | 386 (85.4) | 247 (86.1) |  |
| Missing | 202 (9.1) | 97 (6.5) | 65 (14.4) | 40 (13.9) |  |
| **Malaria Prophylaxis** | | | | |  |
| zero doses | 8(0.4) | 6 (0.5) | 0 | 2 (0.8) | p= 0.006 |
| One-two doses | 600 (29.6) | 395 (28.6) | 146 (35.5) | 59 (25.0) |  |
| more than 3 doses | 1,418(69.9) | 978 (70.9) | 265 (64.5) | 175 (74.2) |  |
| Missing | 198(9.0) | 106 (7.2) | 41 (9.1) | 51 (17.8) |  |
| **Tetanus Prophylaxis** | | | | |  |
| Not given | 2 (0.1) | 2 (0.2) | 0 | 0 |  |
| 1 dose | 347 (16.5) | 216 (15.2) | 86 (20.3) | 45 (17.8) | p= 0.265 |
| 2 doses | 1,321 (62.8) | 912 (63.9) | 253 (59.8) | 156 (61.7) |  |
| 3 or more doses | 433 (20.6) | 297 (20.8) | 84 (19.9) | 52 (20.6) |  |
| Missing information | 121 (5.5) | 58 (4.0) | 29 (6.5) | 34 (11.9) |  |
| **Ultrasound done** | | | | |  |
| Before 24 weeks | 21 (5.2) | 5 (2.9) | 10 (5.5) | 6 (11.5) |  |
| After 24 weeks | 381 (94.8) | 163 (97.0) | 172 (94.5) | 46 (88.5) | p= 0.265 |
| Missing | 1,822 (82.0) | 1,317 (88.7) | 270 (59.8) | 235 (81.9) |  |
| **Haemoglobin (g/dl) at last ANC visit** | | |  |  |  |
| Normal >=11 g/dl | 970 (52.4) | 695 (52.2) | 167 (54.1) | 108 (51.7) | p = 0.769 |
| Mild Anaemia (8.1-10.9) | 835 (45.1) | 606 (45.5) | 132 (42.7) | 97 (46.4) |  |
| Severe Anaemia (<8g) | 45 (2.4) | 31 (2.3) | 10 (3.2) | 4 (1.9) |  |
| Missing | 374 (16.9) | 153 (10.4) | 143 (31.7) | 78 (27.2) |  |
| Missing refers to missing information/or unknown. All these variables were excluded from the multivariable model due to reasons described in the methods section (for example, excessive missing data, too few elements in the clinically relevant cells, etc.)  The proportional distribution of each variable is calculated as a percentage of women with available data for that specific variable (with the proportion of missing excluded from the denominator), while the proportional distributions of women with missing data for the variable is calculated as a percentage of the total number of women for the outcome regardless of whether data was missing or not. | | | | | |

**UNIVARIABLE AND MULTIVARIABLE RESULTS**

A total of 2,224 study participants were involved in this embedded case control study.

| **Supplementary Table 2: Multivariable* logistic regression for Pre-facility Stillbirths against live healthy newborns** | | | | |
| --- | --- | --- | --- | --- |
| **Variable Category** | **COR (95%CI)** | ***p-value*** | **AOR (95%CI)** | ***p-value*** |
| **Age group (years)** |  |  |  |  |
| 15- 19 years | 0.50 (0.32-0.77) | 0.002 | 0.30 (0.14-0.63) | 0.001 |
| 20 - 35 years | 1 |  | 1 |  |
| 36 - 45 years | 1.46 (1.07-20) | 0.018 | 0.84 (0.46-1.52) | 0.558 |
| **Parity** |  |  |  |  |
| Para 1 | 0.72 (0.58-0.91) | 0.005 | 0.98(0.65-1.48) | 0.936 |
| para 2-4 | 1 |  |  |  |
| Para ≥ 5 | 1.29 (0.85-1.94) | 0.228 | 1.04 (0.49-2.24) | 0.915 |
| **Referral status** |  |  |  |  |
| Self-referred/home | 0.53 (0.35-0.80) | 0.003 | 2.31 (0.88-6.07) | 0.09 |
| Peripheral Health Facilities | 1.89 (1.17-3.03) | 0.009 | 3.84 (1.33-11.09) | 0.013 |
| **ANC risk Factor** |  |  |  |  |
| No | 1 |  |  |  |
| Yes | 2.68 (2.02-3.56) | 0 | 2.60 (1.47-4.58) | 0.001 |
| **Admission danger sign** |  |  |  |  |
| None | 1 |  |  |  |
| One or more danger sign | 9.75 (7.63-12.45) | 0 | 4.66 (2.97-7.31) | 0 |
| **Stage of labour** |  |  |  |  |
| latent phase (0-3 cms) | 1.77 (1.34-2.34) | 0 | 1.31 (0.81-2.11) | 0.276 |
| Early active phase (4-6cms) | 1 |  |  |  |
| late active labour >6 cms | 1.39 (1.05-1.83) | 0.02 | 1.24 (0.81-1.92) | 0.325 |
| **Status of liquor on admission** |  |  |  |  |
| Intact membranes | 1 |  |  |  |
| Clear liquor | 0.47 (0.33-0.66) | 0 | 0.38 (0.24-0.61) | 0 |
| Meconium liquor | 6.38 (4.16-9.8) | 0 | 5.36 (2.79-10.29) | 0 |
| **Partograph use** |  |  |  |  |
| Action line not crossed | 1 |  |  |  |
| Crossed action line | 1.38 (0.89-2.12) | 0.147 | 2.25 (1.21-4.18) | 0.011 |
| **Induction of labour** |  |  |  |  |
| No induction of labour |  |  |  |  |
| Induction of labour | 5.36 (3.56-8.07) | 0 | 4.42 (2.08-9.41) | 0 |
| **Mode of delivery** |  |  |  |  |
| Spontaneous vaginal delivery | 1 |  |  |  |
| Vacuum assisted birth | 1.05 (0.34-3.26) | 0.939 | 1.10 (0.19-6.31) | 0.917 |
| Caesarian Section | 0.67 (0.51-0.89) | 0.005 | 0.14 (0.07-0.28) | 0 |
| Breech | 9.41(3.02-29.36) | 0 | 12.12 (2.73-53.78) | 0.001 |
| **Second Stage of Labour** |  |  |  |  |
| No challenges encountered | 1 |  |  |  |
| second stage challenge documented | 11.52 (8.63-15.36) | 0 | 5.57(3.24-9.58) | 0 |
| **Birthweight** |  |  |  |  |
| 2000- 2499 | 4.40 (3.13-6.18) | 0 | 5.03 (2.95-8.57) | 0 |
| 2500- 3000 | 1 |  |  |  |
| 3000- 3499 | 0.40 (0.3-0.53) | 0 | 0.41 (0.26-0.63) | 0 |
| 3500- 3999 | 0.38 (0.26-0.56) | 0 | 0.35 (0.19-0.64) | 0.001 |
| Greater than 4000 | 1.17 (0.72-1.89) | 0.524 | 0.91 (0.36-2.29) | 0.843 |
| **Hypertensive disorders in Pregnancy** | |  |  |  |
| Normal blood pressure |  |  |  |  |
| Any hypertension (mild-severe-PEE) | 4.72 (3.3-6.76) | 0 | 2.85 (1.44-5.67) | 0.003 |
| **Blood pressure measurement on admission**** |  |  |  |  |
| Normal Blood Pressure | 1 |  |  |  |
| Mild hypertension | 1.93 (1.42-2.62) | 0 | 1.79 (1.27-2.52 | 0.001 |
| Severe hypertension | 3.04 (1.83-5.06) | 0 | 2.11 (1.19-3.73 | 0.01 |

*Adjusted for all variables in the table except Blood pressure on admission

****** This was a sub-analysis: adjusted for age of mother, parity and birthweight of baby, this variable was not included in the main multivariable analysis.

| **Supplementary Table 3: Multivariable* logistic regression for Intra-facility perinatal deaths (intrafacility stillbirths and early pre discharge neonatal deaths) compared to live healthy newborns** | | | | |
| --- | --- | --- | --- | --- |
| **Variable category** | **COR (95%CI)** | ***p-value*** | **AOR (95%CI)** | ***p-value*** |
| **Age group (years)** |  |  |  |  |
| 15-19 | 0.70 (0.44-1.12) | 0.135 | 0.45 (0.18-1.13) | 0.089 |
| 20-35 | 1 |  |  |  |
| 36-45 | 1.42 (0.97-2.07) | 0.069 | 0.89 (0.42-1.89) | 0.76 |
| **Parity** |  |  |  |  |
| Para 1 | 0.89 (0.69-1.16) | 0.389 | 1.02 (0.6-1.76) | 0.933 |
| Para 2-4 | 1 |  |  |  |
| Para ≥ 5 | 0.68 (0.36-1.28) | 0.236 | 0.99 (0.37-2.65) | 0.978 |
| **Referral status** |  |  |  |  |
| Self-referred/home | 0.60 (0.36-0.98) | 0.04 | 0.86 (0.3-2.48) | 0.783 |
| Study HFs | 1 |  |  |  |
| Peripheral Health facilities | 1.30 (0.73-2.31) | 0.382 | 1.05 (0.32-3.47) | 0.934 |
| **ANC risk Factor** |  |  |  |  |
| No | 1 |  |  |  |
| Yes | 2.40 (1.71-3.37) | 0 | 3.70 (1.96-6.98) | 0 |
| **Admission danger sign** |  |  |  |  |
| None | 1 |  |  |  |
| One or more danger sign | 3.09 (2.29-4.18) | 0 | 1.51 (0.76-2.99) | 0.236 |
| **Stage of labour** |  |  |  |  |
| Latent phase | 2.28 (1.62-3.22) | 0 | 1.22 (0.68-2.2) | 0.499 |
| Early active phase (4-6cms) | 1 |  |  |  |
| Late active labour >6 cms | 1.16 (0.8-1.68) | 0.445 | 1.16 (0.65-2.07) | 0.618 |
| **Status of liquor on admission** |  |  |  |  |
| Intact membranes | 1 |  |  |  |
| Clear liquor | 0.47 (0.33-0.66) | 0 | 1.12 (0.65-1.91) | 0.688 |
| Meconium liquor | 6.38 (4.16-9.8) | 0 | 4.44 (1.86-10.57) | 0.001 |
| **Partograph use** |  |  |  |  |
| Action line not crossed | 1 |  |  |  |
| Crossed action line | 4.97 (3.37-7.34) | 0 | 4.16 (2.29-7.56) | 0 |
| **Induction of labour** |  |  |  |  |
| No induction of labour | 1 |  |  |  |
| Induction of labour | 3.42 (2.06-5.68) | 0 | 2.74 (0.97-7.72) | 0.056 |
| **Mode of delivery** |  |  |  |  |
| Spontaneous vaginal delivery | 1 |  |  |  |
| Vacuum assisted birth | 7.83 (3.45-17.76) | 0 | 6.23 (1.65-23.55) | 0.007 |
| Caesarian Section | 2.59 (1.97-3.4) | 0 | 0.93 (0.53-1.63) | 0.801 |
| Breech | 33.29 (11.05-100.30) | 0 | 40.30 (8.75-185.61) | 0 |
| **Second Stage of Labour** |  |  |  |  |
| No challenges encountered | 1 |  |  |  |
| second stage challenge documented | 11.52 (8.63-15.36) | 0 | 20.04 (12.02-33.41) | 0 |
| **Birthweight** |  |  |  |  |
| 2000- 2499 | 3.96 (2.61-6.03) | 0 | 5.57 (2.62-11.84) | 0 |
| 2500- 3000 | 1 |  |  |  |
| 3000- 3499 | 0.81 (0.59-1.11) | 0.182 | 0.76 (0.43-1.35) | 0.353 |
| 3500- 3999 | 0.61 (0.39-0.93) | 0.023 | 0.44 (0.2-0.94) | 0.034 |
| Greater than 4000 | 1.23 (0.67-2.26) | 0.505 | 1.36 (0.49-3.82) | 0.556 |
| **Any Hypertensive disorders in Pregnancy** | |  |  |  |
| Normal blood pressure | 1 |  |  |  |
| Any hypertension (mild-severe-PEE) | 2.77 (1.75-4.38) | 0 | 2.90 (1.03-8.14) | 0.043 |
| **Blood pressure measurement on admission **** |  |  |  |  |
| Normal Blood Pressure | 1 |  |  |  |
| Mild moderate hypertension | 1.39 (0.91-2.1) | 0.124 | 0.80 (0.36-1.78) | 0.585 |
| Severe hypertension | 1.27 (0.56-2.9) | 0.569 | 1.37 (0.32-5.77) | 0.669 |

*Adjusted for all variables in the table except Blood pressure on admission

** This was a sub-analysis: adjusted for age of mother, parity and birthweight of baby (not included in the main multivariable model)
