# Supplementary material for: Incidence and determinants of perinatal mortality in five urban hospitals in Dar es Salaam, Tanzania: a cohort study with an embedded case–control analysis
Source: BMC Pregnancy Childbirth. 2024 Jan 13;24:62. doi: 10.1186/s12884-023-06096-1 (PMC10787400; doi:10.1186/s12884-023-06096-1)
Supplement: Supplementary file 2 — Additional file 2. Perinatal case control study data collection tool. [file 12884_2023_6096_MOESM2_ESM.pdf]

CCBRT (English (en))

Variable Name	Question Text	Saved Value
q01	01. Facility Name	11 Amana Hosp<br>22 Mbagala Rangitatu Hosp<br>33 Mwananyamala Hosp<br>44 Temeke Hosp<br>55 Sinza Hosp
q01_1	01.1. Is this a Double Entry?	0 Yes<br>1 No
q02	02. Data entry clerk Code	User entered integer
q02_1	02.1. Data entry clerk file SN number (Of the files you have entered so far, what number is this file?)	User entered integer
q03_1	03.1. Registration Number	User entered text
q03_2	03.2. Delivery Number	User entered text
c0	Hidden from user	
c1	Hidden from user	
c2	Hidden from user	
PID	Patient ID	User entered text
PID_DoubleEntry	Patient ID	User entered text
q04	04. Is the file a CONTROL file?	0 Yes<br>1 No
note1	###General Information	User entered text
q05_check	05a. Is the Date and time of admission indicated in the file?	1 Yes - Both Indicated<br>2 No - Both Not-indicated<br>3 Only Date Indicated<br>4 Only Time indicated
q05	05. Date of admission	User selected date
q06	06. Time of admisison	User selected time

q07	07. Age of mother (Years)	User entered integer
q08	08. Admitted from /referred from:	1 Came directly from home<br>2 RCH clinic of same hospital as place of birth<br>3 Antenatal ward of same hospital as place of delivery<br>4 Referred patient from other Health Facility
q08a	08a. Choose Facility	1 Sinza Hospital<br>2 Mwananyamala hospital<br>3 Amana hospital<br>4 MNH<br>5 Mbagala Rangitatu Hosp<br>6 Mnazimoja Hosp<br>7 Chanika Hospital<br>8 Pugu HC<br>9 Buguruni HC<br>10 Kimara HC<br>11 Mbagala Roundtable HC<br>12 Yombo Vituka Disp<br>13 Majimatitu disp<br>14 Tandika<br>15 Vikindu<br>16 Buza<br>17 Charambe<br>18 Buza<br>19 Consolate RCH<br>20 Chamazi<br>21 Bunju<br>22 Mbezi<br>23 Kigogo HC<br>24 Tandale Disp

		25 Tegeta Disp<br>26 Kunduchi<br>27 Kawe<br>28 Mwenge<br>29 Kigamboni HC<br>30 Vijibweni Hospital<br>96 Other (Specify)
q08_other	Other, specify:	User entered text
q09	09. Parity after this child birth.	User entered decimal
q10	010. Any previous stillbirth/Neonatal death	0 Yes<br>1 No
q11	011. Is the Last mentrual Period (LMP) indicated in the file	0 yes<br>1 No<br>99 Unknown LMP<br>88 Not recorded
q12	012. If yes, write LMP (as written in the case notes)	User selected date
q13_check	013a. Is the Expected Date of Delivery date indicated in the file?	0 Yes<br>1 No<br>88 Not recorded
q13	013. EDD (as written in case notes)	User selected date
q14	014. Gestation age at admission(as written in the case file)	User entered decimal
q15	015. Was Ultrasound conducted at any time during pregnancy	0 Yes<br>1 No<br>88 Not recorded
q16	016. Gestation age when the 1st Ultrasound was done	User entered decimal
q17_check	17a.Is the date when Ultra Sound was done indicated in the file?	0 Yes<br>1 No<br>88 Not recorded

q17	017. Date when FIRST documented Ultrasound done	User selected date
note2	###Antenatal Care (ANC)	User entered text
q19	019. Number of ANC visits	User entered integer
q20_check	020. Is the Date of last ANC visit indicated?	0 Yes<br>1 No<br>2 RCH 4 card not available<br>88 Not recorded
q21	021. Date of last ANC visit	User selected date
q22	022. Any ANC risk factor	0 No risk factor indicated in case notes<br>1 One Previous CS<br>2 Two or morePrevious CS<br>3 Previous still birth or neonatal death<br>4 More than 3 previous abortion<br>5 Less than 150 cms height<br>6 More than ten years since previous childbirth<br>7 Excess bleeding in previous pregnancy/postpartum<br>8 Heart disease<br>9 Tuberculosis<br>10 Diabetes<br>11 Retained placenta in previous pregnancy<br>12 Deformity of pelvis/CPD<br>96 Others (specify)
q22_check	It is not possible to select "No risk factor indicated in case notes" together with other options. Please go back and correct the selection. 	User entered text
q22_other	Other, specify:	User entered text

q23	023. Systolic Blood pressure at last ANC visit (mmHg)	User entered decimal
q24	024. Diastolic Blood pressure at Last ANC visit (mmHg)	User entered decimal
q25	025. Woman's HIV status at ANC:	1 HIV Positive<br>2 Negative<br>88 Not recorded
q26	026. If HIV positive write treatment	0 On ART<br>1 Not on treatment<br>88 Missing Information/Not indicated
q27	027. Number of IPT (Intermttent presumptive Treatment) malaria doses	User entered integer
q28	028. Tetanus Toxoid number of doses in index pregnancy	User entered integer
q29	029. VDRL (For Syphilis) test performed	1 Yes<br>2 Not done<br>88 Not recorded
q30	030. VDRL test results (if done)	1 Reactive<br>2 Non reactive
q31	031. Haemoglobin level (at last clinic visit)-from case file (g/dl)	User entered decimal
q32_check	32a. Is the date of last Haemoglobin test indicated in the case file?	0 Yes<br>1 No<br>88 Not recorded
q32	032. Date of last Haemoglobin- from case file	User selected date
note3	###First examination during labor/on admission 	User entered text
q33_check	33a. Is the date and time for first examination indicated in the file?	1 Yes - Both Indicated<br>2 No - Both Not-indicated<br>3 Only Date Indicated<br>4 Only Time indicated
q33	033. Date of first examination	User selected date

q34	034. Time of first examination	User selected time
q35	035. Maternal Systolic Blood Pressure on admission - (mmHg) (or first examination)	User entered decimal
q35a	035a. Maternal Systolic Blood Pressure above 160, What actions were taken?	User entered text
q36	036. Maternal Diastolic Blood pressure on admission - (mmHg)	User entered decimal
q36a	036a. Maternal Diastolic Blood pressure above 110, What actions were taken?	User entered text
q37	037. Maternal Pulse on admission (beats per minute)	User entered decimal
q37a	037a. Maternal Pulse, What actions were taken?	User entered text
q38	038. Maternal Respiratory rate on admssion (per min)	User entered decimal
q38a	038a. If maternal respiratory rate of less than 12 or greater than 30. What action was taken?	User entered text
q39	039. Maternal temperature on admission ('C)	User entered decimal
q39a	039a. Maternal temperature above 38, What actions were taken?	User entered text
q40	040. Fetal heart rate on admission (beats per minute)	User entered decimal
q40a	040a. Fetal heart rate was (<110 or >160), What actions were taken?	User entered text
q41_check	041a. If no fetal heart heard on admission, Is the date last recorded/heard indicated in the file?	0 Yes<br>1 No<br>88 Not recorded
q41	041. If no fetal heart heard on admission, write approximate date last recorded/ heard	User selected date
q42	042. Cervical dilation on first assessment/admission (cm)	User entered decimal
q43	043. state of membranes on admission	0 Intact<br>1 Ruptured membranes - Clear liquor<br>2 Ruptured membranes - Blood stained<br>3 Ruptured membranes - Meconium stained<br>4 Ruptured membranes - Liquor not described<br>88 Not recorded
q44_check	044a. Is the date and time of rupture of membranes that occurred during labour/delivery, indicated in the file??	1 Yes - Both Indicated<br>2 No - Both Not-indicated<br>3 Only Date Indicated

		4 Only Time indicated
q44	044. For those with Intact membranes on admission, Write date of rupture of membranes	User selected date
q45	045. For those with Intact membranes on admission, Write time of rupture of membranes	User selected time
q46	046. Type of rupture	0 Spontaneous rupture (SRM)<br>1 Artificial rupture (ARM)<br>88 Not indicated
q47	047. State of liquor during labor	0 Not documented<br>1 Ruptured - Clean<br>2 Ruptures - Blood stained<br>3 Ruptured - Meconium stained
q48	048. Admission danger signs / conditions	0 No danger signs<br>1 Headache<br>2 Blurred vision<br>3 Chest pain<br>4 Difficulty in breathing<br>5 Cough<br>6 Severe abdominal Pain<br>7 Vaginal bleeding<br>8 Fever (≥38.0 C)<br>9 Shivering<br>10 Vomiting<br>11 General body weakness/feels unwell<br>12 Tiredness<br>13 Low hemoglobin<br>14 Convulsions<br>15 Unconciousness

		16 Calf pain<br>17 Fetal distress or fetal heart (<110 or >160) beats per minute<br>18 Loss of fetal movements<br>19 Decreased fetal movements<br>20 Vaginal discharge-foul smelling<br>21 Draining- premature rupture of membranes<br>22 Oxygen saturation (<90)<br>23 Blood pressure (> 140/90 mmHg)
q48_check	It is not possible to select "No danger signs" together with other options. Please go back and correct the selection. 	User entered text
q048a	048a. If yes to any of the danger signs, specify what actions were taken:	User entered text
note4	###Second vaginal examination during labor	User entered text
q49	049. Was the Second examination ever conducted	0 Yes<br>1 No
q50_check	050a. Is the date and time for Second examination indicated in the file?	1 Yes - Both Indicated<br>2 No - Both Not-indicated<br>3 Only Date Indicated<br>4 Only Time indicated
q50	050. Second exam date	User selected date
q51	051. Second exam_ time	User selected time
q52	052. Second examination of cervical dilatation (cm)	User entered decimal
q53	053. Second exam Systolic Blood pressure - (mmHg)	User entered decimal
q53a	053a. Second exam Systolic Blood pressure above 160, What actions were taken?	User entered text
q54	054. Second exam Diastolic Blood pressure (mmHg)	User entered decimal
q54a	054a. Second exam Diastolic Blood pressure above 110, What actions were taken?	User entered text
q55	055. Second exam Maternal pulse rate (beats per minute)	User entered decimal

q56	056. Second examination Maternal temperature ('c)	User entered decimal
q56a	056a. Second examination Maternal temperature above 38, What actions were taken?	User entered text
q57	057. Second examination fetal heart rate (beats per minute)	User entered decimal
q57a	057a. Second examination fetal heart rate was (<110 or >160), What actions were taken?	User entered text
note5	###Third Vaginal examination during labor	User entered text
q58	058. Was the Third examination ever conducted	0 Yes<br>1 No
q59_check	059a. Is the date and time for Third examination indicated in the file?	1 Yes - Both Indicated<br>2 No - Both Not-indicated<br>3 Only Date Indicated<br>4 Only Time indicated
q59	059. Third exam date	User selected date
q60	060. Third exam_ time	User selected time
q61	061. Third examination of cervical dilatation (cm)	User entered decimal
q62	062. Third exam Systolic Blood pressure - (mmHg)	User entered decimal
q62a	062a. Third exam Systolic Blood pressure above 160, What actions were taken?	User entered text
q63	063. Third exam Diastolic Blood pressure (mmHg)	User entered decimal
q63a	063a. Third exam Diastolic Blood pressure above 110, What actions were taken?	User entered text
q64	064. Third exam Maternal pulse rate (beats per minute)	User entered decimal
q65	065. Third examination Maternal temperature ('c)	User entered decimal
q65a	065a. Third examination Maternal temperature above 38, What actions were taken?	User entered text
q66	066. Third examination fetal heart rate (beats per minute)	User entered decimal
q66a	066a. Third examination fetal heart rate was (<110 or >160), What actions were taken?	User entered text
note6	###Fourth Vaginal examination during labor	User entered text
q67	067. Was the Fourth examination ever conducted	0 Yes<br>1 No
q68_check	068a. Is the date and time for Fourth examination indicated in the file?	1 Yes - Both Indicated<br>2 No - Both Not-indicated<br>3 Only Date Indicated

		4 Only Time indicated
q68	068. Fourth exam date	User selected date
q69	069. Fourth exam_ time	User selected time
q70	070. Fourth examination of cervical dilatation (cm)	User entered decimal
q71	071. Fourth exam Systolic Blood pressure - (mmHg)	User entered decimal
q71a	071a. Fourth exam Systolic Blood pressure above 160, What actions were taken?	User entered text
q72	072. Fourth exam Diastolic Blood pressure (mmHg)	User entered decimal
q72a	072a. Fourth exam Diastolic Blood pressure above 110, What actions were taken?	User entered text
q73	073. Fourth exam Maternal pulse rate (beats per minute)	User entered decimal
q74	074. Fourth examination Maternal temperature ('c)	User entered decimal
q74a	074a. Fourth examination Maternal temperature above 38, What actions were taken?	User entered text
q75	075. Fourth examination fetal heart rate (beats per minute)	User entered decimal
q75a	075a. Fourth examination fetal heart rate was (<110 or >160), What actions were taken?	User entered text
note7	###Fifth Vaginal Examination during labor	User entered text
q76	076. Was the Fifth examination ever conducted	0 Yes<br>1 No
q77_check	077a. Is the date and time for Fifth examination indicated in the file?	1 Yes - Both Indicated<br>2 No - Both Not-indicated<br>3 Only Date Indicated<br>4 Only Time indicated
q77	077. Fifth exam date	User selected date
q78	078. Fifth exam_ time	User selected time
q79	079. Fifth examination of cervical dilatation (cm)	User entered decimal
q80	080. Fifth exam Systolic Blood pressure - (mmHg)	User entered decimal
q80a	080a. Fifth exam Systolic Blood pressure above 160, What actions were taken?	User entered text
q81	081. Fifth exam Diastolic Blood pressure (mmHg)	User entered decimal
q81a	081a. Fifth exam Diastolic Blood pressure above 110, What actions were taken?	User entered text
q82	082. Fifth exam Maternal pulse rate (beats per minute)	User entered decimal
q83	083. Fifth examination Maternal temperature ('c)	User entered decimal

q83a	083a. Fifth examination Maternal temperature above 38, What actions were taken?	User entered text
q84	084. Fifth examination fetal heart rate (beats per minute)	User entered decimal
q84a	084aFifth examination fetal heart rate was (<110 or >160), What actions were taken?	User entered text
q85	085. Did the patient have 6 or more Pelvic examinations	0 Yes<br>1 No
q86	086. If had 6 or more vaginal exams	User entered text
q87	087. Was the Action-line crossed	0 Yes<br>1 No<br>88 Not recorded
note8_1	###Induction of Labour/Augmentation 	User entered text
q88	088. Did the patient have induction of labour	0 Yes<br>1 No
q89	089. What method was used for induction	1 Oxytocin/pitocin<br>2 Misoprostol<br>3 vagiprost<br>4 Dinoprostol<br>5 Artificial rupture of membranes<br>6 Baloon catheter
q90	090. Did the patient have augumentation of labour, that is the use of oxytocin after labour onset?	0 Yes<br>1 No
q91	091. If yes to augumentation, then write Cervical dilation when oxytocin AUGMENTATION started (cm)	User entered decimal
q92	092. If yes to augumentation, then what was number and strength of Contraction when oxytocine WAS STARTED	0 No Contraction<br>1 Mild contractions (less than 20s)<br>2 Moderate contractions (between 20s and 35s)<br>3 Strong contractions (more

		than 40sec and more than 3 in 10 minutes)
q93	093. If yes to augumentation, then write Fetal heart rate when oxytocin started (beats per minute)	User entered decimal
note8	###Delivery 	User entered text
q94_check	094a. Is the date and time for delivery indicated in the file?	1 Yes - Both Indicated<br>2 No - Both Not-indicated<br>3 Only Date Indicated<br>4 Only Time indicated
q94	094. Date of delivery	User selected date
q95	095. Time of delivery	User selected time
q96	096. Mode of delivery	0 Spontaneous Vaginal Delivery<br>1 Vacuum extraction<br>2 Caesarian Section (CS)<br>3 Breech<br>88 Not recorded/Missing<br>96 Other
q96_other	Other, specify	User entered text
q97	097. If yes to CS, Was Cesarean Section elective or emergency	0 Emergency<br>1 Elective<br>88 Not recorded
q98	098. If yes to CS, Indicate indication for CS	User entered text
q99_check	099a. If Emergency CS, Is the date and time when decision for CS was made indicated in the file?	1 Yes - Both Indicated<br>2 No - Both Not-indicated<br>3 Only Date Indicated<br>4 Only Time indicated
q99	099. If Emergency CS , indicate date when decision for CS was made	User selected date
q100	100. If Emergency CS, indicate time when decision for CS was made	User selected time

q101_check	101a. If Emergency CS, Is the date and time the CS operation was performed indicated in the file?	1 Yes - Both Indicated<br>2 No - Both Not-indicated<br>3 Only Date Indicated<br>4 Only Time indicated
q101	101. If Emergency CS, indicate Date the CS operation was performed	User selected date
q102	102. If Emergency CS, indicate time the CS operation was performed	User selected time
q103	103. Indicate if there were any challenges in the second stage	0 No challenges encountered<br>1 Second stage duration greater than 2 hours<br>2 Cord around the neck/knot<br>3 Stuck head in breech presentation<br>4 Shoulder dystocia<br>5 Fetal destress<br>88 Not recorded<br>96 Other
q103_check	It is not possible to select "No challenges encountered" or "Not recorded/missing " together with other options. Please go back and correct the selection. 	User entered text
q103_other	Other, specify	User entered text
note8_2	###Birth outcome and management to the neonate	User entered text
q104	104. Baby birthweight (grams)	User entered decimal
q105	105. Status of baby at birth	0 Born Alive<br>1 Fresh Still birth<br>2 Macerated still birth<br>88 Not recorded
q106	106. Apgar score_at one minute	User entered decimal
q107	107. Apgar score_at 5 minutes	User entered decimal
q108	108. Was Neonatal resuscitation done	0 No resuscitation<br>1 Stimulation<br>2 Suction done

		3 Bag and mask<br>88 Not recorded
q108_check	It is not possible to select "No resuscitation" or "Not recorded" together with other options. Please go back and correct the selection. 	User entered text
q109	109. If baby born alive AND not a control and needed resuscitation or noted to be sick, indicate any management given to the baby	0 Not documented<br>1 10% dextrose<br>2 Oxygen therapy<br>3 Vital sign monitoring<br>4 Tube feeding<br>5 Medications like phenobarbitone<br>6 Medications like antibiotics<br>96 Other (specify)
q109_check	It is not possible to select "Not documented" together with other options. Please go back and correct the selection. 	User entered text
q109_other	Other, specify	User entered text
q110	110. Final status of the baby on discharge (outcome measure)	0 Healthy baby, Apgar Score 9/10 and discharged home alive. (control)<br>1 Still birth (pre facility) - No fetal heart heard on admission<br>2 Intra facility Still Birth - had fetal heart on admission/labour<br>3 Neonatal death - Born alive and then baby died
q111_check	111a. Is the date and time of fetal heart last heard/recorded indicated in the file?	1 Yes - Both Indicated<br>2 No - Both Not-indicated<br>3 Only Date Indicated<br>4 Only Time indicated
q111	111. For all admissions that had fetal heart during admission to the ward, write the	User selected date

<table>
<tr><td></td><td>date fetal heart was last heard</td><td></td></tr>
<tr><td>q112</td><td>112. For all admissions that had fetal heart during admission to the ward, write the time fetal heart was last heard</td><td>User selected time</td></tr>
<tr><td>q113</td><td>113. Total number of times Fetal heart recorded in case notes</td><td>User entered integer</td></tr>
<tr><td>q114</td><td>114. If baby born alive and then died indicate where did the baby die?</td><td><table><tr><td>1</td><td>Labor ward</td></tr><tr><td>2</td><td>Post natal ward</td></tr><tr><td>3</td><td>Neonatal ward</td></tr><tr><td>88</td><td>Not recorded</td></tr></table></td></tr>
<tr><td>q115_check</td><td>115. Is the date and time of neonatal death (Born alive and then baby died) indicated in the file?</td><td><table><tr><td>1</td><td>Yes - Both Indicated</td></tr><tr><td>2</td><td>No - Both Not-indicated</td></tr><tr><td>3</td><td>Only Date Indicated</td></tr><tr><td>4</td><td>Only Time indicated</td></tr></table></td></tr>
<tr><td>q115a</td><td>115a. If baby born alive and then died indicate date of neonatal death</td><td>User selected date</td></tr>
<tr><td>q115b</td><td>115b. If the baby was born alive and then died, write the main complaint of the baby.</td><td>User entered text</td></tr>
<tr><td>q115c</td><td>115c. If the baby was born alive and then died, write investigations that were done and result if available.</td><td>User entered text</td></tr>
<tr><td>q115d</td><td>115d. If the baby was born alive and then died, write the management/treatment that was given.</td><td>User entered text</td></tr>
<tr><td>q116</td><td>116. If baby born alive and then died indicate time of neonatal death</td><td>User selected time</td></tr>
<tr><td>note9</td><td>###Other management to the Mother during labour?</td><td>User entered text</td></tr>
<tr><td>q117</td><td>117. What was the status for Protein in urine during labour?</td><td><table><tr><td>1</td><td>Checked - Negative</td></tr><tr><td>2</td><td>Checked - Trace</td></tr><tr><td>3</td><td>Checked - 1+</td></tr><tr><td>4</td><td>Checked - 2+</td></tr><tr><td>5</td><td>Checked - 3+</td></tr><tr><td>88</td><td>Not recored</td></tr></table></td></tr>
<tr><td>q118</td><td>118. What additional investigations were done during labour and delivery?</td><td><table><tr><td>1</td><td>Hemoglobin</td></tr><tr><td>2</td><td>Ultrasound</td></tr><tr><td>3</td><td>Blood for grouping and cross matching</td></tr><tr><td>4</td><td>Serum creatinine</td></tr></table></td></tr>
</table>

		5 Bed side clotting time<br>6 Liver function test<br>88 None<br>96 Others (specify)
q118_check	It is not possible to select "Not Recorded" together with other options. Please go back and correct the selection. 	User entered text
q118_1a	118.1. Hemoglobin Results	User entered text
q118_2a	118.2. Ultrasound Results	User entered text
q118_3a	118.3. Blood for grouping and cross matching Results	User entered text
q118_4a	118.4. Serum creatinine Results	User entered text
q118_5a	118.5. Bed side clotting time Results	User entered text
q118_6a	118.6. Liver function test Results	User entered text
q118_other	Other, specify name of investigation:	User entered text
q118_other_results	${0} Results:	User entered text
q119	119. If Blood pressure (systolic blood pressure elevated above 139 MmHg or diastolic blood pressure above 89 mmHg (HDP)or Eclampsia, Was observation & Treatment sheet used?	0 Yes<br>1 No<br>88 Not recorded
q120	120. Indicate organ symptoms?	1 Patient had No symptoms<br>2 Headache<br>3 Blurred vission<br>4 Abdominal pain<br>5 Convulsions<br>88 Not recorded<br>96 Others (specify)
q120_other	Other, specify:	User entered text
q121	121. if signs of HDP, or eclampsia, Was Patellar reflex recorded (at least once)?	0 Yes<br>1 No<br>88 Not recorded
q122	122. Was IV fluids given before delivery (Normal Saline/Ringers lactate/DNS)	0 Yes

		1 No<br>88 Not recorded
q122_1	122.1. Type of fluid used	User entered text
q122_2	122.2. How much fluid was given	User entered text
q123	123. Was Fluid Balance (input-output chart) used ?	0 Yes<br>1 No
q124	124. Was blood given	0 Yes<br>1 No
q125	125. If yes to blood given - When was blood given	1 Before delivery<br>2 After delivery<br>88 Not recorded
q126	126. Any other medications/treatment /drugs given to the mother during Labour/before delivery	0 None<br>1 Oxytocin<br>2 Any other oxytocic (specify type and amount)<br>3 Amoxicillin<br>4 Ampiclox<br>5 Gentamycin<br>6 Metronidazole<br>7 Ceftriaxone<br>8 Hydrocortisone<br>9 Dexamethasone<br>10 Aldomet<br>11 Nifedipine<br>12 Magnesium Sulphate<br>13 Medications prescribed by the doctor but not recorded in the chart<br>96 Others (specify)

q126_check	It is not possible to select "None" together with other options. Please go back and correct the selection. 	User entered text
q126_1a	126.1. Oxytocin Dosage	User entered text
q126_2a	126.2a. Other oxytocic type	User entered text
q126_2b	126.2b. Other oxytocic Dosage amount	User entered text
q126_3a	126.3. Amoxicillin Dosage	User entered text
q126_4a	126.4. Ampiclox Dosage	User entered text
q126_5a	126.5. Gentamycin Dosage	User entered text
q126_6a	126.6. Metronidazole Dosage	User entered text
q126_7a	126.7. Ceftriaxone Dosage	User entered text
q126_8a	126.8. Hydrocortisone Dosage	User entered text
q126_9a	126.9. Dexamethasone Dosage	User entered text
q126_10a	126.10. Aldomet Dosage	User entered text
q126_11a	126.11. Nifedipine Dosage	User entered text
q126_12a	126.12. Magnesium Sulphate Dosage	User entered text
q126_13a	126.13. Medications prescribed by the doctor but not recorded in the chart, Specify:	User entered text
q126_other	126a. Other, specify name of medications/treatment /drugs given:	User entered text
q126_other_dose	126b. ${1} Dosage and/or Results:	User entered text
q127	127. Any other medications/treatment /drugs given to the mother after delivery?	0 None<br>1 Oxytocin<br>2 Any other oxytocic (specify type and amount)<br>3 Intravenous fluid<br>4 Amoxicillin<br>5 Ampiclox<br>6 Gentamycin<br>7 Metronidazole<br>8 Ceftriaxone<br>9 Hydrocortisone<br>10 Dexamethasone<br>11 Aldomet<br>12 Nifedipine<br>13 Magnesium Sulphate

		14 Medications prescribed by the doctor but not recorded in the chart<br>96 Others (specify)
q127_check	It is not possible to select "None" together with other options. Please go back and correct the selection. 	User entered text
q127_1a	127.1. Oxytocin Dosage	User entered text
q127_2a	127.2a. Other oxytocic type	User entered text
q127_2b	127.2b. Other oxytocic Dosage amount	User entered text
q127_3a	127.3a. Intravenous fluid type	User entered text
q127_3b	127.3b. Intravenous fluid Dosage	User entered text
q127_4a	127.4. Amoxicillin Dosage	User entered text
q127_5a	127.5. Ampiclox Dosage	User entered text
q127_6a	127.6. Gentamycin Dosage	User entered text
q127_7a	127.7. Metronidazole Dosage	User entered text
q127_8a	127.8. Ceftriaxone Dosage	User entered text
q127_9a	127.9. Hydrocortisone Dosage	User entered text
q127_10a	127.10. Dexamethasone Dosage	User entered text
q127_11a	127.11. Aldomet Dosage	User entered text
q127_12a	127.12. Nifedipine Dosage	User entered text
q127_13a	127.13. Magnesium Sulphate Dosage	User entered text
q127_14a	127.14. Medications prescribed by the doctor but not recorded in the chart, Specify:	User entered text
q127_other	127a. Other, specify name of medications/treatment /drugs given:	User entered text
q127_other_dose	127b. ${2} Results:	User entered text
q128	128. Was blood pressure taken after delivery?	0 Yes<br>1 No
q129	129. Total number of times Blood pressure measured after delivery?	User entered integer
q130	130. Any maternal complication during labour & delivery, list?	0 Healthy mother<br>1 Coincidental conditions (accidents,herbal)<br>2 Known patient with heart diseases

		3	Diabetes
		4	Postpartum Uterus with Ruptured uterus
		5	Postpartum Haemorrhage due to uterine atony
		6	Postpartum Haemorrhage due to tears and other causes
		7	Anemia
		8	Pneumocystis pneumonia (PCP)
		9	Urinary Tract Infection (UTI)
		10	Respiratory infection
		11	Malaria
		12	Fever due to other causes
		13	Puerperal Sepsis
		14	Antepartum Obstetric haemorrhage without hypertension
		15	Antepartum Obstetric haemorrhage with hypertension
		16	Chronic Hypertension
		17	Mild Hypertension (BP 140/90-159/89mmhg)
		18	Severe Pre Eclampsia (BP=160/90 mmhg)
		19	Eclampsia (convulsions)
		20	Perineal Tear
		21	Maternal death
		96	Other
q130_20_other	Perineal Tear, Specify:	User entered text	
q130_other	Other, specify:	User entered text	
q131	131. If yes to maternal death, Cause of maternal death:	User entered text	
note9_1	###Partograph Use	User entered text	

q132	132. Use of Partograph	0 Every field filled<br>1 Partially filled (missing observations)<br>2 First cervical dilation 3 cms and above plotted on alert line<br>3 Used incorrectly (filled but no action taken)<br>4 Not used at all<br>5 Not applicable
q132_check	It is not possible to select "Not used at all" or "Not applicable" together with other options. Please go back and correct the selection. 	User entered text
note10	###Management post delivery	User entered text
q133_check	133a. Is the discharge date of the Mother indicated in the file?	0 Yes<br>1 No<br>88 Not recorded
q133	133. Discharge date of the Mother	User selected date
note10_1	###Final Comments	User entered text
q134	134. Any substandard care to mother or baby/any additional notes in the file relevant to the care received.	0 None<br>1 Free text<br>88 Not recorded
q134_other	Free Text:	User entered text
q135	135. Was the perinatal death audited	0 Yes<br>1 No - no form available for review<br>2 Not applicable (for control)
q136	136. If perinatal audit done, write Primary obstetric cause of death code as written in the audit form	User entered text
q137	137. If perinatal audit done, write avoidable factor as written in the audit form	User entered text
meta	Hidden from user	
instanceID	Hidden from user	

instanceName	Hidden from user	
